# High-Performance Memristive Synapse Based on Space-Charge-Limited Conduction in LiNbO_3_

**DOI:** 10.3390/nano14231884

**Published:** 2024-11-23

**Authors:** Youngmin Lee, Sejoon Lee

**Affiliations:** 1 Division of System Semiconductor, Dongguk University, Seoul 04620, Republic of Korea; ymlee@dongguk.edu; 2 Quantum-Functional Semiconductor Research Center, Dongguk University, Seoul 04620, Republic of Korea

**Keywords:** LiNbO_3_, oxygen vacancy migration, memristive effect, electronic synapse

## Abstract

Advancing neuromorphic computing technology requires the development of versatile synaptic devices. In this study, we fabricated a high-performance Al/LiNbO_3_/Pt memristive synapse and emulated various synaptic functions using its primary key operating mechanism, known as oxygen vacancy-mediated valence charge migration (V_O_-VCM). The voltage-controlled V_O_-VCM induced space-charge-limited conduction and self-rectifying asymmetric hysteresis behaviors. Moreover, the device exhibited voltage pulse-tunable multi-state memory characteristics because the degree of V_O_-VCM was dependent on the applied pulse parameters (e.g., polarity, amplitude, width, and interval). As a result, synaptic functions such as short-term memory, dynamic range-tunable long-term memory, and spike time-dependent synaptic plasticity were successfully demonstrated by modulating those pulse parameters. Additionally, simulation studies on hand-written image pattern recognition confirmed that the present device performed with high accuracy, reaching up to 95.2%. The findings suggest that the V_O_-VCM-based Al/LiNbO_3_/Pt memristive synapse holds significant promise as a brain-inspired neuromorphic device.

## 1. Introduction

Recent advances in information and intelligence technologies, such as the Internet of Things, big data analysis, data-intensive image process, and artificial intelligence, have significantly increased the demand for novel electronic devices that enable fast and efficient data computation [1,2]. The conventional von Neumann architecture is anticipated to encounter inherent limitations due to its bottleneck effect, which arises from serial data processing and high power consumption. This bottleneck is primarily due to the separation of data processing units and memory units in von Neumann computing architectures [3,4]. To address this critical issue, neuromorphic computing devices have garnered substantial interest. Neuromorphic computing aims to replicate the functionality of the human brain, particularly in processing, storing, and transmitting data in parallel [5,6]. The parallel processing capability of neuromorphic computing allows simultaneous data computation across multiple interconnected nodes, which can effectively mimic the neural networks of the human brain. This can lead to exceptional performance in complex data processing, pattern recognition, and autonomous learning, with remarkable power efficiency [7,8].

In biological neural networks, data processing occurs through the modulation of synaptic plasticity, which connects multiple neurons [6,9,10]. The memristive behaviors of analog memristors closely mimic the key functionalities of biological synapses. Specifically, memristors exhibit voltage-controlled dynamic changes in electrical conductance as well as nonvolatile data retention [11]. This allows analog memristors to act as electronic synapses capable of expressing electronic data in multi-level conductance states across a large dynamic range, enabling synaptic weight updates with high linearity and symmetry and ensuring spatiotemporal variability with fluctuation [1,12]. These characteristics enable analog memristors to mimic the learning capabilities of biological synapses. Consequently, various in-memory architectures, known as memristive synapses, have been demonstrated based on several memristive switching mechanisms, including the electromigration of valence charges (e.g., defect charges [13,14] and metal ions [15,16]), electrochemical metallization [17,18], phase transitions [19,20], ferroelectric polarization [21,22], and redox reactions in organic materials [23,24]. Among these, oxygen vacancy (V_O_)-mediated valance charge migration (VCM) in oxide materials is particularly advantageous. The electric field-controlled V_O_-VCM not only allows reversible filamentary switching but also enables fine-tuning of resistance levels [25,26]. In essence, the degree of V_O_-VCM can be precisely controlled by adjusting the parameters of the applied voltage pulses to the device (e.g., polarity, amplitude, width, and interval) [27].

To demonstrate V_O_-VCM-based memristive synapses, various oxide materials such as HfO_2_ [28,29,30], TiO_2_ [31,32], WO_3_ [33,34], Ta_2_O_5_ [27,35], and LiNbO_3_ [36,37] have garnered significant attention due to their intrinsic point defects, diverse growth methods, valence charge control techniques, and excellent resistive switching characteristics. Among these oxide materials, LiNbO_3_ stands out for its potential to achieve uniform analog switching, owing to its oxygen octahedron structure [38,39]. In rhombohedral LiNbO_3_, oxygen atoms share faces along the polar trigonal axis, and these oxygen octahedra are interspersed with Li and Nb atoms. This arrangement provides four pathways along the edges of the octahedron, allowing for easy migration of V_O_ within the lattice [40,41]. Given these intrinsic advantages, LiNbO_3_-based synaptic devices have recently attracted considerable interest [42,43,44,45,46,47,48]. As noted earlier, the degree of V_O_-VCM directly influences the synaptic characteristics of memristive devices. Consequently, the V_O_-VCM behavior in LiNbO_3_ can effectively emulate synaptic characteristics, such as the linear and symmetric potentiation/depression of synaptic weights [36,37,44] as well as spike-timing-dependent synaptic plasticity [42]. To enhance the V_O_-VCM properties in LiNbO_3_, several techniques have been recently proposed and demonstrated to control the V_O_ density in single-crystalline LiNbO_3_. For example, methods like crystal ion slicing using low-energy Ar^+^ irradiation [42,43,44,45,46] and locally tailored strain doping through He^+^ or H^+^ ion implantation are effective for controlling the V_O_ density in LiNbO_3_ [47,48]. However, despite the previse V_O_ control offered by these techniques, they complicate the device fabrication process. Therefore, a simpler, more straightforward method is needed to fabricate V_O_-VCM-mediated LiNbO_3_ memristors. For future applications in artificial neural networks, it is essential to develop a memristive synapse array in a crossbar architecture that utilizes a simplified fabrication process. In this context, directly growing LiNbO_3_ onto the electrode material is crucial.

In this work, we investigate the facile fabrication of simple V_O_-VCM-based Au/LiNbO_3_/Pt memristive synapses and characterize their synaptic characteristics. The top-to-bottom Au/LiNbO_3_/Pt devices were fabricated by directly sputtering LiNbO_3_ onto the Pt bottom electrode, followed by the formation of an Al top electrode onto the LiNbO_3_ active layer. Here, we report the effects of LiNbO_3_ growth temperature on the material properties and their corresponding synaptic characteristics in V_O_-VCM-based Au/LiNbO_3_/Pt memristors. To provide insight into the device operation, the charge transport mechanisms are also thoroughly analyzed and discussed in detail.

## 2. Experimental Details

**Figure 1:**
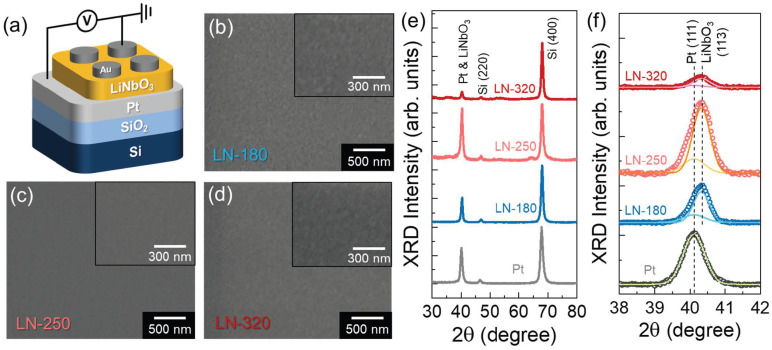
(**a**) Schematic of the Al/LiNbO_3_/Pt memristive synapse. Surface FE-SEM images of the (**b**) LN-180, (**c**) LN-250, and (**d**) LN-320 layers grown on (111) Pt/SiO_2_/Si substrates at different temperatures of 180, 250, and 320 °C, respectively. (**e**) Wide-angle XRD patterns of the LN-180, LN-250, and LN-320 samples. (**f**) Deconvoluted XRD pattern at the Bragg angle of ~40.1°, showing the portions of (111) Pt and (113) LiNbO_3_ phases. The insets in (**b**–**d**) show the zoomed-in view of each sample.

Figure 1a shows the fabricated device structure of the top-to-bottom contact two-terminal Al/LiNbO_3_/Pt memristor. First, a Ti adhesion layer (≈3 nm thick) was deposited by D.C. sputtering at 450 °C onto the SiO_2_/Si substrate to enhance adhesion between the Pt bottom electrode and the substrate. Subsequently, a 120 nm thick, mirror-like Pt (111) layer was deposited onto the Ti adhesion layer via D.C. sputtering at 500 °C. Next, a 50 nm thick LiNbO_3_ layer was grown at 180–320 °C on the Pt/SiO_2_/Si substrate using R.F. magnetron sputtering with an R.F. power of 80 W. During the 60 min LiNbO_3_ deposition, the working pressure was maintained at 25 mTorr, while a gas mixture of Ar (12 sccm) and O_2_ (6 sccm) was continuously supplied. Finally, circular Al top electrodes (100 µm in diameter) were formed onto the LiNbO_3_ layers.

The surface morphology of the LiNbO_3_ layers was monitored using field-emission scanning electron microscopy (FE-SEM) with a Hitachi S4800 electron microscope (Tokyo, Japan). Crystallographic structures and lattice phases were analyzed via X-ray diffraction (XRD) using a Bruker D8 Advance (Madison, WI, USA) with a Cu K*α*_1_ radiation source. The valence states of the LiNbO_3_ components were examined using X-ray photoelectron spectroscopy (XPS) with a Thermos Fisher Scientific ESCALab250Xi system (Waltham, MA, USA). The ferroelectric properties of the LiNbO_3_ layers were evaluated using polarization vs. voltage (P–V) measurements with a Precision RT66C Ferroelectric Tester (Radiant, Albuquerque, NM, USA). The electrical characteristics and synaptic functions of the Al/LiNbO_3_/Pt memristor were assessed using a B1500A/B1530A semiconductor parameter analyzer (Keysight, Santa Rasa, CA, USA).

## 3. Results and Discussion

In thin-film devices, the homogeneity of crystal grains is crucial for maintaining stable on-state current flow because crystalline defects such as grain boundaries and pits can increase leakage current, potentially leading to device failure. To investigate the effect of growth temperature on the film texture, we deposited three different LiNbO_3_ layers at 180, 250, and 320 °C and assessed their morphological properties. For simplicity, we refer to the samples grown at these temperatures as LN-180, LN-250, and LN-320, respectively. As shown in the FE-SEM image of LN-180 (Figure 1b), the LiNbO_3_ layer grown at the low temperature of 180 °C displayed an inhomogeneous and rough surface. However, when the growth temperature increased to 250 °C, the LN-250 sample exhibited a smooth and well-merged surface (Figure 1c). In contrast, the surface of the LN-320 sample became rough again when the growth temperature increased up to 320 °C (Figure 1d).

The surface morphology is closely related to the crystallographic properties of thin films. Therefore, we performed XRD analysis on the LiNbO_3_ samples. Figure 1e shows the XRD patterns of LN-180, LN-250, and LN-320 layers deposited onto Pt (111)/SiO_2_/Si substrates. In all samples, three predominant XRD peaks were observed at Bragg angles of ~40.1°, ~46.7°, and ~67.8°. The peaks at ~46.6° and ~67.8° are well known to correspond to the (220) and (400) crystal planes of diamond-structured Si [49], while the peak at approximately 40° is associated with both the (111) Pt and (113) LiNbO_3_ phases [50]. As deconvoluted in Figure 1f, the XRD peak at ~40.1° originated from the (111) phase of cubic Pt [51], while that at ~40.3° was attributed to the (113) phase of rhombohedral LiNbO_3_ [52]. According to a previous study by Ono et al. [50], when LiNbO_3_ is grown on a (111) Pt substrate, it tends to increase along preferential orientations perpendicular to the (001) and (113) directions. This suggests that the LiNbO_3_ layers in this study were effectively grown along the rhombohedral (113) phase direction without segregation into Nb_2_O_5_ and LiNb_3_O_8_. When comparing the intensity of the (113) LiNbO_3_ peak, the XRD results correlate well with the FE-SEM images. Specifically, the LN-250 sample exhibited a stronger (113) LiNbO_3_ peak intensity than LN-180, while LN-320 showed a significant degradation in crystallinity. Based on the XRD and FE-SEM analyses, we can conclude that the LiNbO_3_ sample grown at 250 °C is more suitable for fabricating high-quality memristive devices than those grown at other temperatures.

**Figure 2:**
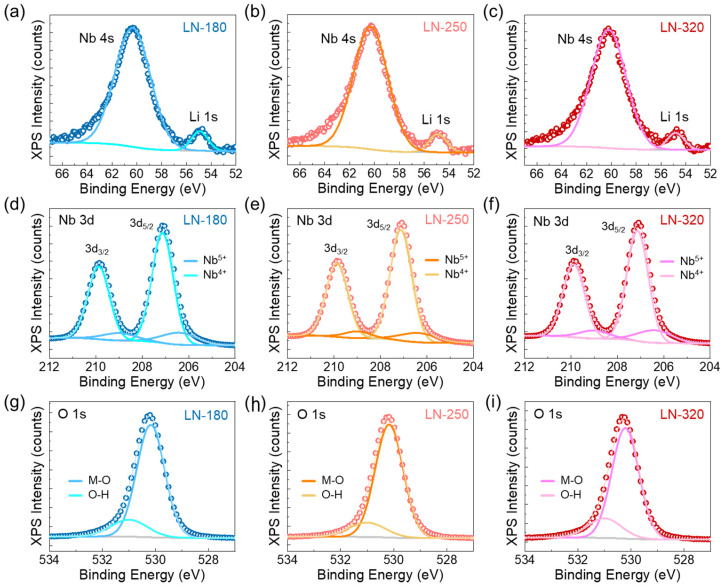
XPS spectra of the LiNbO_3_ layers grown at different temperatures. Li 1s and Nb 4s core levels of (**a**) LN-180, (**b**) LN-250, and (**c**) LN-320. Nb 3d core levels of (**d**) LN-180, (**e**) LN-250, and (**f**) LN-320. O 1s core levels of (**g**) LN-180, (**h**) LN-250, and (**i**) LN-320.

Next, the valence states of the elemental species were investigated through XPS analysis. Figure 2a–c present the Li 1s and Nb 4s core-level spectra of the LiNbO_3_ layers grown at 180–320 °C, respectively. In all samples, distinct peaks were observed for both Li 1s and Nb 4s at 54.9 and 60.3 eV, respectively.

Regardless of the growth temperature, there were no significant changes in the peak positions or the intensity ratio between Li 1s to Nb 4s, as seen in Figure 2a–c. This indicates that the stoichiometric composition of Li and Nb remained nearly identical across the LN-180, LN-250, and LN-320 samples [53,54]. To further explore the valence states of Nb, high-resolution XPS measurements were performed for the Nb 3d core level. Figure 2d–f show that the Nb 3d spectrum can be deconvoluted into two distinct components: Nb^5+^ and Nb^4+^. The doublet peaks of 3d_5/2_ at 209.8 eV and 3d_3/2_ at 207.1 eV correspond to Nb^5+^ [54,55], while additional doublet peaks at 209.1 eV (3d_5/2_) and 206.4 (3d_3/2_) represent Nb^4+^. The presence of Nb^4+^ in LiNbO_3_ is closely related to the formation of V_O_, which compensates for two electrons within the Nb site [56,57]. Eventually, V_O_ acts as a donor within the LiNbO_3_ lattice [58]. The existence of V_O_ was further confirmed through the O 1s core-level spectra, as shown in Figure 2g–i, where two characteristic oxygen bonds are evident: Nb-O-Li at 530.1 eV and V_O_ at 531 eV [53].

**Figure 3:**
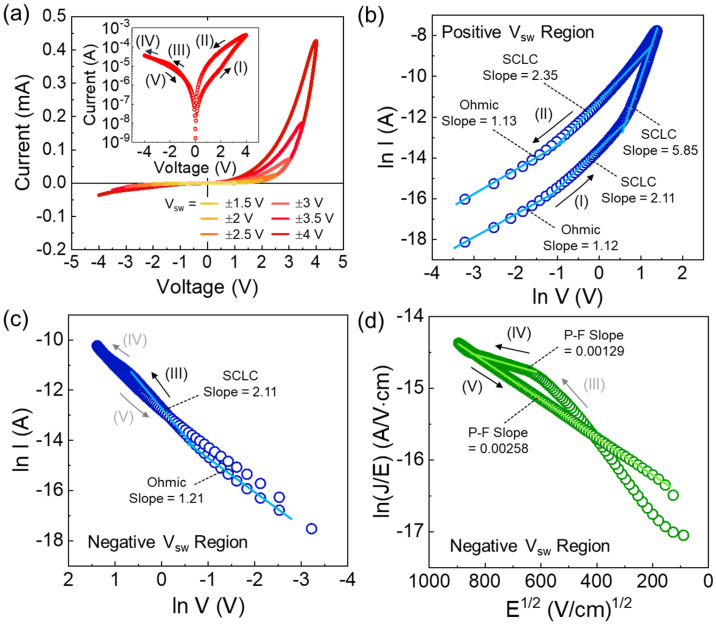
(**a**) I–V characteristic curves of the Al/LiNbO_3_/Pt memristive synapse (LN-250) measured under various *V*_sw_ ranges. SCLC plots in the (**b**) positive and (**c**) negative *V*_sw_ regions. (**d**) P-F plot in the negative *V*_sw_ region. The inset in (**a**) illustrates the hysteretic behavior represented by the semi-logarithmic I–V curve.

As previously discussed, V_O_ plays a crucial role in facilitating the VCM-based memristive switching behavior in LiNbO_3_. Therefore, we evaluated the current–voltage (I–V) characteristics of the Al/LiNbO_3_/Pt memristors. It was observed that the I–V characteristics varied depending on the morphological properties of LiNbO_3_. Particularly, the memristors fabricated with LN-320 exhibited unstable and leaky I–V curves, while the devices using LN-180 and LN-250 demonstrated stable memristive switching characteristics (see Figure S1). However, when the sweep voltage (*V*_sw_) exceeded ±3 V, the LN-180 device also showed unstable I–V behaviors with sudden glitches (Figure S1a–c). Based upon these results, we accordingly focused further electrical characterization on the LN-250 sample. As shown in Figure 3a, the LN-250 memristor clearly revealed voltage polarity-dependent asymmetric hysteresis loops (see the inset of Figure 3a). Moreover, both the memory window and on-state current increased progressively with increasing *V*_sw_. The device demonstrated robust self-rectifying memristive characteristics [42,43,44,45,46,47,48], which are advantageous for controlling the linear and symmetric potentiation/depression of synaptic weights [36,44] and for suppressing sneak path currents during the depression process [59,60].

In V_O_-VCM-based memristors, the memristive switching behaviors can be attributed to two primary mechanisms. The first involves the migration and redistribution of V_O_, leading to changes in electrical conductance by forming V_O_ channels, resulting in filamentary conduction (i.e., memristive switching via filamentary conduction) [36,45]. The second mechanism is the gradual change in the on-state current, mediated by V_O_-VCM, which modulates the potential barrier at the electrode/oxide interface (i.e., memristive switching via interfacial barrier modulation) [44,46,61]. To gain further insight into the observed switching behavior of the LN-250 memristor, we analyzed its conduction mechanism using the space-charge-limited conduction (SCLC) model [62,63], which is associated with the V_O_-VCM behavior in oxide materials [43,44,45,46]. The I–V relationship for SCLC conduction is given by the following:

(1) $$J_{SCLC}=\frac{9}{8}\varepsilon_{i}\mu \theta \frac{V^{2}}{d^{3}},$$

where *ε_i_* is the static dielectric constant of the oxide, *μ* is the carrier mobility, *θ* is the ratio of free carrier density to trapped charge density, and *d* is the oxide thickness.

In Region I (see inset in Figure 3a), when a low positive voltage (*V*_sw_ > 0) was applied to the device, the current increased linearly with the applied voltage (i.e., slope ≈ 1.12). As the magnitude of *V*_sw_ further increased, the current followed Child’s law with a slope of approximately 2.11 (i.e., I ∝ V^2^). After this point, the slope sharply increased to 5.85, indicating that the high-electric field created a temporary conductive area region, corresponding to trap-limited SCLC [36,45]. Upon returning to the lower *V*_sw_ region in Region II (see inset in Figure 3a), the current followed Child’s law again, with a slope of approximately 2.35, consistent with the trap-filled SCLC mechanism [46]. In the negative *V*_sw_ region (Region III, Figure 3c), the slope was found to be 2.11, also consistent with the trap-limited SCLC mechanism. However, in Region IV, at higher negative voltages, the slope changed, indicating that charge transport shifted to a different mechanism than SCLC. To identify the appropriate mechanism in Region IV, we replotted and analyzed the I–V curve using several transport models, such as Poole–Frenkel (P-F) emission, Fowler-Northeim tunneling, and Schottky emission. Then, we found that the P-F emission model provided the best fit to the measured I–V curve (see Figure S2). According to the literature [48,64], P-F emission is predominantly governed by the trap-limited bulk conduction mechanism, given by the following:

(2) $$J_{PF}=q\mu N_{c}Eexp\left[\frac{-q\left(\phi_{T}-\sqrt{qE/\pi \varepsilon_{0}\varepsilon_{r}}\right)}{kT}\right],$$

where *q* is the elementary charge, *μ* is the electronic drift mobility, *N_c_* is the density of states in the conduction band, *E* is the electric field, *k* is a Boltzmann constant, *ϕ_T_* is the trap energy level, *ε_0_* is the permittivity of free space, and *ε_r_* is the dielectric constant of the material. From this, the slope in the P-F plot can be given as follows:

(3) $$Slope=m{\left(\frac{q^{3}}{\pi \varepsilon_{0}\varepsilon_{r}{\left(kT\right)}^{2}}\right)}^{1/2},$$

where *m* is the constant that distinguishes the main conduction mechanism. For example, *m* = 1 for P-F emission, and *m* = 2 for shallow traps [64,65]. From the ln(*J*/*E*) vs. *E*^1/2^ plot (Figure 3d), two distinct slopes were observed: 0.00129 and 0.00258 in Regions IV and V, respectively. Since the refractive index (*ε_r_*^1/2^) of LiNbO_3_ is reported to be 2.28 in the literature [43,48,66], the value of *m* in Region IV was found to be unity. This suggests that P-F emission dominates the charge conduction in Region IV. Similarly, the *m* value in Region V was found to be 2, indicating that shallow trap-mediated P-F emission governs the conduction in this region.

**Figure 4:**
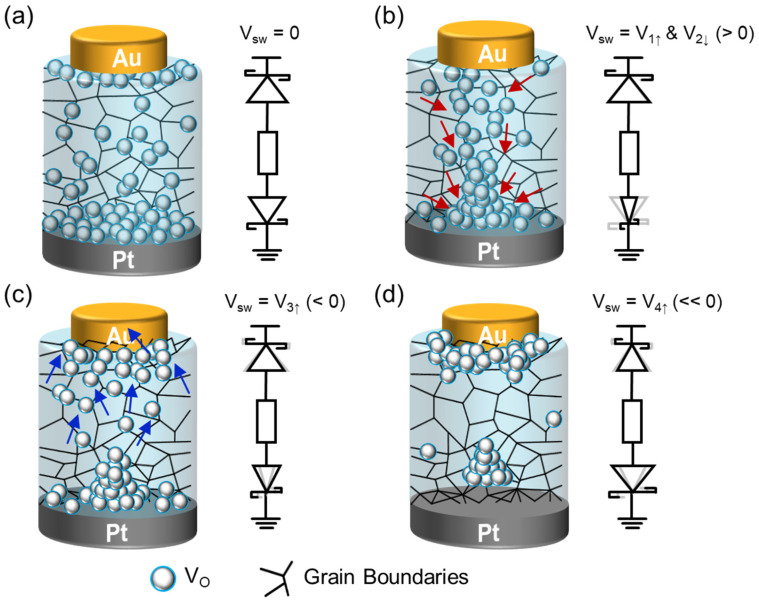
V_O_-VCM behaviors in the Al/LiNbO_3_/Pt memristive synapse at (**a**) *V*_sw_ = 0 V, (**b**) *V*_sw_ = *V*_1↑_ and *V*_2↓_ (>0), (**c**) *V*_sw_ = *V*_3↓_, (<0), and (**d**) *V*_sw_ = *V*_4↑_ (<<0).

Based on the above results, we here interpret the plausible charge transport mechanism in the present Au/LiNbO_3_/Pt memristor. Figure 4 illustrates the V_O_-VCM-mediated SCLC behavior at various bias voltages. From the XPS results, we assume the existence of V_O_ in the LiNbO_3_ active layer. During the fabrication of the Au/LiNbO_3_/Pt device, the LiNbO_3_ layer was grown directly onto the Pt metallic electrode. Consequently, a large amount of V_O_ is likely to be distributed at the bottom region of LiNbO_3_ near the Pt electrode, as the high density of grain boundaries forms in the initial LiNbO_3_ layer deposited on the Pt electrode [45]. At zero bias (Figure 4a), the potential barrier at the LiNbO_3_/Pt interface (i.e., Schottky-like barrier at the metal/oxide interface) is expected to be slightly lower due to the reduction in electrochemical potential caused by V_O_ [43,46]. Similarly, the potential barrier at the Au/LiNbO_3_ interface is also reduced, as abundant V_O_ is generated during the final growth stage of LiNbO_3_ that resides underneath the Au top electrode. As shown on the right side of Figure 4a, the fabricated Au/LiNbO_3_/Pt device thus acts like a two-diode-connected resistor. Here, it should be noted that the exact origin of the Schottky-like potential barrier remains unclear. However, prior studies [43,44,45,46,67,68,69,70] have observed rectifying behaviors at metal/LiNbO_3_ interfaces (e.g., Au, Cr, Pt, and Ti), likely due to V_O_-induced Fermi-level pinning [69,70], which contributes to the formation of Schottky-like barriers.

When a positive bias (*V*_sw_ = *V*_1↑_ > 0) is applied with the Pt electrode grounded, V_O_ migrates (i.e., V_O_-VCM) toward the Pt electrode along the grain boundaries (Figure 4b), resulting in the formation of localized V_O_ clusters (i.e., V_O_ group) near the Pt interface due to vacancy–vacancy interactions [71,72]. Simultaneously, the charges from the migrating V_O_ contribute to trap-controlled SCLC within the LiNbO_3_ active layer (e.g., Region I). As V_O_ clusters near the Pt electrode, they reduce the local electrochemical potential, further lowering the potential barrier at the LiNbO_3_/Pt interface [43,47]. This process sustains a high on-state current at a relatively high bias voltage (*V*_sw_). The on-state current persists until the V_O_ clusters are redistributed by applying a negative *V*_sw_. Therefore, the high on-state current remains even when the *V*_sw_ decreases to a lower voltage (e.g., *V*_sw_ = *V*_2↓_ < *V*_1↑_ in Region II), leading to memristive hysteresis in the I–V characteristics of the Au/LiNbO_3_/Pt device.

After switching the voltage (*V*_sw_) to the negative *V*_3↑_ (Figure 4c), the clustered V_O_ groups begin to disintegrate, allowing V_O_ to migrate toward the Au/LiNbO_3_ interface. This initiates trap-controlled SCLC at this bias state (e.g., Region III). It is important to note that the density of migrated V_O_ will not increase further, even with the application of a higher negative *V*_sw_. This is because V_O_ clusters near the LiNbO_3_/Pt interface tend to remain stable. Specifically, since V_O_ tends to stabilize in its neutral valence state (V_O_^0^) [73], the density of electromigrating V_O_ is limited. Hence, the charge transport mechanism changes from trap-controlled SCLC to P-F emission, characterized by minimal current flow (e.g., Region IV). When a more negative voltage (*V*_sw_ = *V*_4↑_ << 0) is applied, the conducting path is abruptly disconnected due to the rupture of [47] localized V_O_ clusters at the LiNbO_3_/Pt interface (Figure 4d). Consequently, the potential barrier at the LiNbO_3_/Pt interface significantly increases, allowing only a small current to flow through the shallow trap-mediated P-F emission (e.g., Region V). Therefore, this type of Au/LiNbO_3_/Pt memristor exhibits the rectified asymmetric hysteresis characteristics.

**Figure 5:**
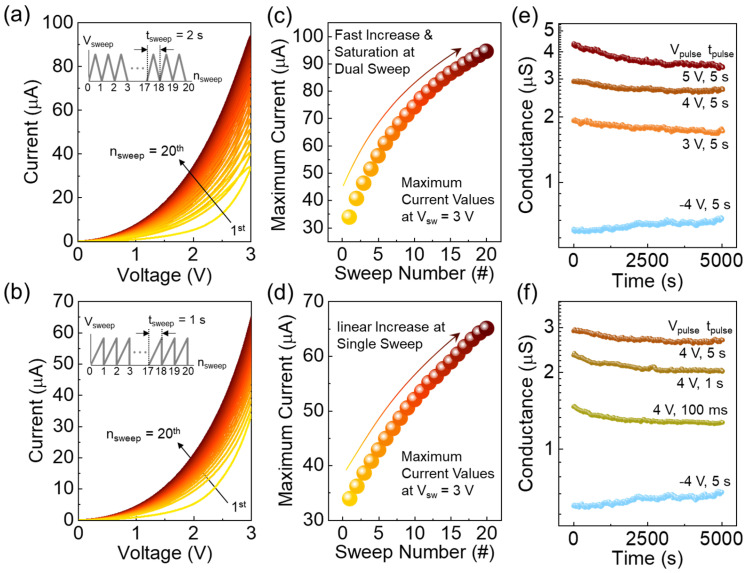
I–V characteristic curves measured over 20 consecutive voltage sweeps at *V*_sw_ = 0–3 V performed by the (**a**) dual-sweep mode and the (**b**) single-sweep mode. Maximum current evolution as a function of *n*_sw_ for the (**c**) dual-sweep and (**d**) single-sweep modes. Retention characteristics at quadruple states demonstrated by changing the (**e**) magnitude of *V*_pro_ and the (**f**) value of *t*_pro_.

The V_O_-VCM-mediated potential barrier modulation presents an opportunity to emulate synaptic functions because multiple memristive states with varying on-state current levels can be achieved by adjusting the potential barrier at both the Au/LiNbO_3_ and LiNbO_3_/Pt interfaces. To explore this, we examined the synaptic functions of the LN-250 memristor. First, we evaluated the dependence of memristive hysteresis characteristics on the number of voltage sweeps (*n*_sw_). Figure 5a,b show the evolution of the on-state current observed after applying 20 consecutive voltage sweeps with a dual-sweep mode and a single-sweep mode, respectively. For the dual-sweep mode with a sweep time (*t*_sweep_) of 2 s (see inset in Figure 5a), the device clearly exhibited the hysteresis loops, while the maximum current increased rapidly and tended to saturate as the *n*_sw_ increased (see also Figure 5c). In the case of the single-sweep mode with a *t*_sweep_ of 1 s (see inset in Figure 5b), similarly, the maximum current increased with increasing the *n*_sw_ (see also Figure 5d). These indicate that the LN-250 memristor could demonstrate data accumulation in response to the number of consecutive voltage biases (i.e., cumulative learning behavior). Additionally, the device displayed the stable retention characteristics of the multilevel conductance states, which are essential for demonstrating the synaptic functions. As shown in Figure 5e, the device exhibited tenacious data retention characteristics for multiple memory states. Namely, four clear multilevel states, which had been performed by applying voltage pulses with pulse amplitudes (*V*_pulse_) of +5, +4, +3, and −4 V, were tenaciously maintained after 5000 s (Figure 5e). Similarly, as can be seen from Figure 5f, four different tenacious memory states were also achieved by changing the pulse width (*t*_pulse_).

**Figure 6:**
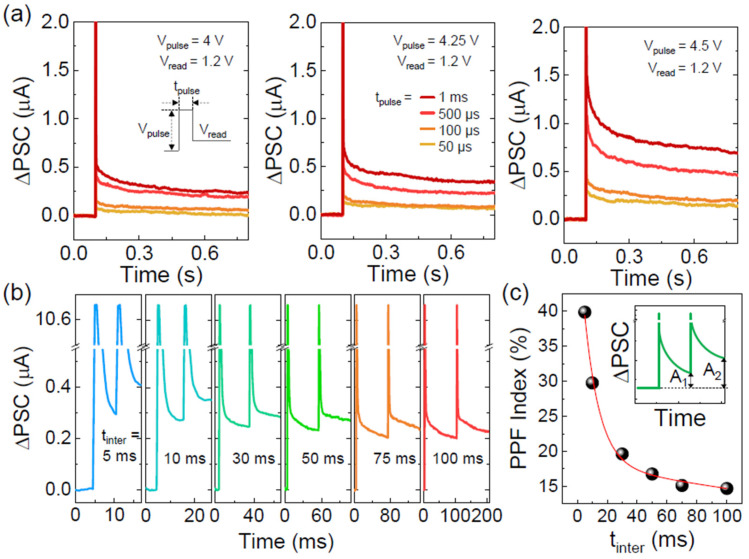
Basic synaptic characteristics of the Al/LiNbO_3_/Pt memristive synapse (LN-250). (**a**) EPSC functions performed at different *V*_pulse_ (4–4.5 V) with different *t*_pulse_ (50 μs–1 ms). (**b**) Dependence of PPF characteristics on *t*_inter_, where *V*_pulse_, *t*_pulse_, and *V*_read_ were fixed at 4 V, 500 μs, and 1.2 V, respectively. (**c**) PPF index as a function of *t*_inter_.

These basic learning behaviors and tenaciously retainable multi-states characteristics are evident for the synaptic activity of the Au/LiNbO_3_/Pt memristor. To examine the synaptic functionality, firstly, we thus measured the excitatory postsynaptic current (EPSC) characteristics. Figure 6a displays the EPSC transient curves of the LN-250 memristor, measured at a read-out voltage (*V*_read_) of 1.2 V after applying a single voltage pulse with varying *V*_pulse_ and *t*_pulse_. When a single voltage pulse (i.e., a presynaptic stimulus) was applied to the device, the electric pulse-stimulated postsynaptic current (∆PSC) stabilized rapidly after an initial decay. Notably, the magnitude of the retained ∆PSC depended on both *V*_pulse_ and *t*_pulse_. For instance, when *V*_pulse_ = 4 V (left panel of Figure 6a), the residual ∆PSC increased with longer *t*_pulse_. Furthermore, the device demonstrated a *V*_pulse_-dependent enhancement of ∆PSC, with greater ∆PSC values observed at *V*_pulse_ = 4.5 V (right panel of Figure 6a) compared to *V*_pulse_ = 4 and 4.25 V. These behaviors are similar to biological synapses, where synaptic plasticity depends on the duration and strength of the stimuli. Thus, it can be inferred that applying consecutive stimuli with moderate *V*_pulse_ and *t*_pulse_ gradually strengthens the synaptic plasticity, enabling the LN-250 memristor to mimic biological synaptic functions.

The above hypothesis can be tested by examining the short- and long-term-memory (STM/LTM) characteristics. As a first step, we evaluated the paired pulse facilitation (PPF) characteristics to investigate the short-term enhancement of synaptic strength. PPF measures the cumulative ∆PSC when two consecutive stimuli are applied. The interval between the two pulses (*t*_inter_) is critical for determining PPF activity because the ∆PSC triggered by the second pulse plays a key role for updating the synaptic weight from its previous state. To assess PPF, we measured the ∆PSC values as a function of *t*_inter_, which varied from 5 to 100 ms, while keeping *t*_pulse_, *V*_pulse_, and *V*_read_ constant at 500 μs, 4 V, and 1.2 V, respectively. Similar to the EPSC characteristics, the PPF curves exhibited typical transient behavior in response to the applied voltage pulses. However, in the case of PPF, the residual ∆PSC value increased following the second pulse (Figure 6b), indicating that the synaptic weights were enhanced from the initial ∆PSC triggered by first pulse to the updated ∆PSC state induced by the second pulse. Notably, as *t*_inter_ increased, the updated ∆PSC values significantly decreased, leading to a weakening of data retention. This is likely due to the diffusion of grouped V_O_ clusters into the bulk region during *t*_inter_ period, driven by concentration gradients [73]. Furthermore, the difference between the first pulse-initiated and second pulse-updated ∆PSC values (*A*_2_ − *A*_1_) decreased exponentially with increasing *t*_inter_. Consequently, the PPF index ((*A*_2_ − *A*_1_)/*A*_1_ × 100%) also showed an exponential decay as a function of *t*_inter_ (Figure 6c). This *t*_inter_-dependent PPF decay can be attributed to two distinct phases of synaptic weight relaxation [74,75]:

(4) $$PF\;index=C_{1}\exp\left(-t/\tau_{1}\right)+C_{2}\exp\left(-t/\tau_{2}\right),$$

where *C*_1_ and *C*_2_ are the initial PPF values for the rapid and slow relaxation phases, respectively; *τ*_1_ and *τ*_2_ are the time constants associated with these two phases, respectively. By fitting the experimental data to Equation (4) (shown as the red line in Figure 6c), *τ*_1_ and *τ*_2_ of the LN-250 memristor were estimated to be 10.09 and 434.08 ms, respectively. In biological synapses, the fast relaxation time enables producing a temporally enhanced synaptic response by short-interval stimuli through rapid resetting of synaptic response. In contrast, the slow relaxation time supports long-term synaptic plasticity even with prolonged intervals in between repeated stimuli [76]. These time constants of biological synapses differ, depending on the characteristics of various synapses (e.g., neurotransmittances, receptor properties, and synaptic roles) [77,78]. Among biological synapses that are responsible for the learning action, the rapid relaxation time typically ranges from a few milliseconds to tens of milliseconds, while the slow relaxation time persists from a hundred milliseconds to a few seconds [79,80]. Therefore, it can be surmised that the present Au/LiNbO_3_/Pt memristor may effectively replicate the basic synaptic functions of biological synapses.

**Figure 7:**
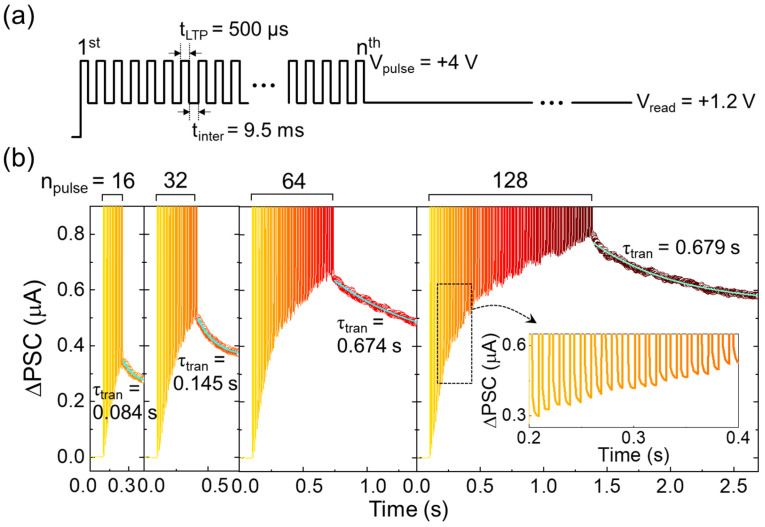
STM-to-LTM transition characteristics of the Al/LiNbO_3_/Pt memristive synapse (LN-250). (**a**) Applied pulse scheme. (**b**) Dependence of potentiation and data retention characteristics on the number of applied pulses. The inset in (**b**) shows a zoomed-in view of the ΔPCS transient curves.

In biological synapses, the transition from STM to LTM plays a fundamental role in synaptic learning. STM temporarily updates the memory state, with the corresponding synaptic weight rapidly reverting to its initial state. In contrast, LTM represents a semipermanent change in synaptic weight, achievable through the application of a large number of consecutive stimuli. This is akin to the rehearsal ability of the human brain [74,81], which can enhance the STM-to-LTM transition probability through repetitive practices. Such a rehearsal action can also be demonstrated in the Au/LiNbO_3_/Pt memristor. After selecting the pulse parameters (i.e., *V*_pulse_ = 4 V, *t*_pulse_ = 500 μs, and *t*_inter_ = 9.5 ms) based on multiple assessments of varying key pulse parameters (see Figure S3), we investigated the STM-to-LTM transition behavior, i.e., rehearsal activity, as a function of the number of applied pulses (*n*_pulse_ = 16, 32, 64, and 128). As shown in Figure 7, consecutive potentiation pulses led to a sequential update of the synaptic weight. Notably, the device exhibited a strong dependence on both the updated synaptic weight and its retention characteristics as a function of *n*_pulse_. Specifically, the consecutive potentiation pulses facilitated an increase un ΔPSC values as *n*_pulse_ increased. Furthermore, the transient time (*τ*_tran_) of the updated ΔPSC also increased from 0.084 to 0.679 s as *n*_pulse_ was increased from 16 to 128, respectively. These results indicate that the device supports STM-to-LTM transition activity, which is characteristic of synaptic learning and memory functions. This STM-to-LTM transition in the Au/LiNbO_3_/Pt memristor can be attributed to V_O_-VCM-mediated potential barrier modulation. As discussed earlier, applying a positive bias voltage promotes the V_O_-VCM behavior within the LiNbO_3_ active layer. Consequently, the degree of V_O_-VCM increases with consecutive voltage pulses, leading to enhanced V_O_ clusterization. This, in turn, increases SCLC in the LiNbO_3_ active layer and reduces the potential barrier at the LiNbO_3_/Pt interface. Moreover, the strong V_O_ clusterization results in robust retention of the hysteretic memory state. Thus, both ΔPSC and *τ*_tran_ increase as *n*_pulse_ increases, enabling the effective STM-to-LTM transition in the Au/LiNbO_3_/Pt memristor.

**Figure 8:**
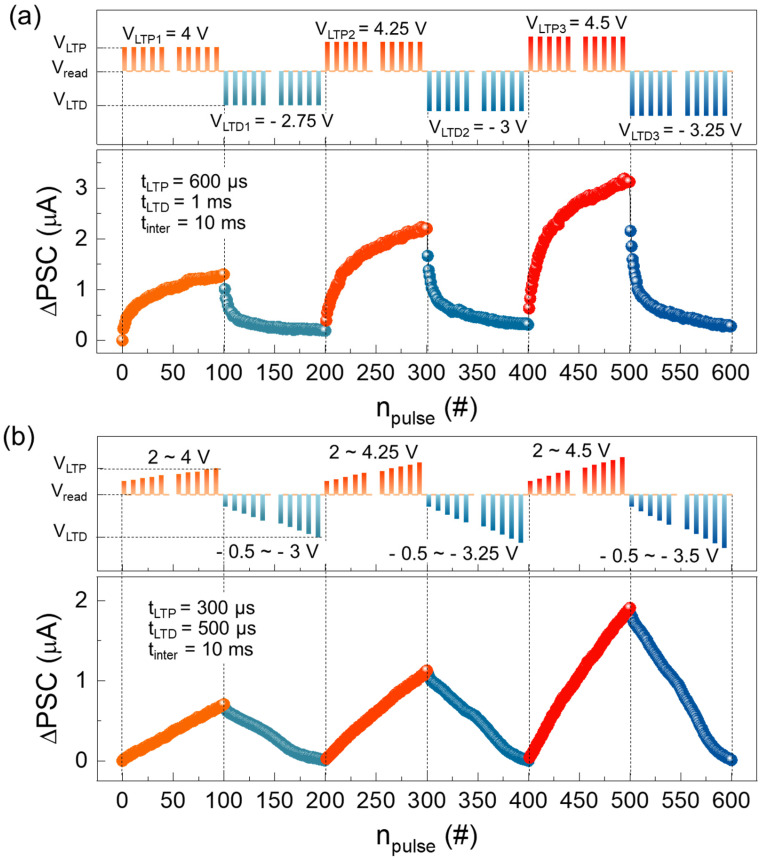
LTP and LTD characteristics of the Al/LiNbO_3_/Pt memristive synapse (LN-250) measured under (**a**) identical and (**b**) incremental pulse schemes. The upper and lower panels in each figure show the applied pulse scheme and the measured LTP/LTD data, respectively.

After observing the STM-to-LTM transition, we examined the long-term potentiation (LTP) and long-term depression (LTD) characteristics by applying continuous 100 LTP and 100 LTD pulses (i.e., *V*_LTP_ and *V*_LTP_). To evaluate the dependence of the ΔPSC dynamic range on the applied *V*_pulse_ magnitude, we varied both *V*_LTP_ and *V*_LTP_ amplitudes, while other parameters were fixed at *t*_LTP_ = 600 μs, *t*_LTD_ = 1 ms, *t*_inter_ = 10 ms, and *V*_read_ = 1.2 V (see the upper panel of Figure 8a). As shown in Figure 8a, the dynamic range of ΔPSC increased with both *V*_LTP_ and *V*_LTP_. For high learning accuracy and efficient training in the electronic synapse, both a wide dynamic range and good linearity are essential [82]. However, the LN-250 memristor exhibited non-linear and asymmetric LTP/LPD behavior. To improve both the linearity and symmetricity of the LTP/LTD characteristics, pulse modulation techniques such as the pulse magnitude modulation [83,84] and pulse frequency modulation [12,85] have been suggested in the literature. Therefore, we attempted to improve both linearity and symmetricity by using incremental *V*_LTP_ and *V*_LTP_ schemes while keeping other parameters fixed at *t*_LTP_ = 300 μs, *t*_LTD_ = 500 μs, *t*_inter_ = 10 ms, and *V*_read_ = 1.2 V (see the upper panel of Figure 8b). As shown in Figure 8b, both linearity and symmetricity were significantly improved using the incremental pulse scheme.

**Figure 9:**
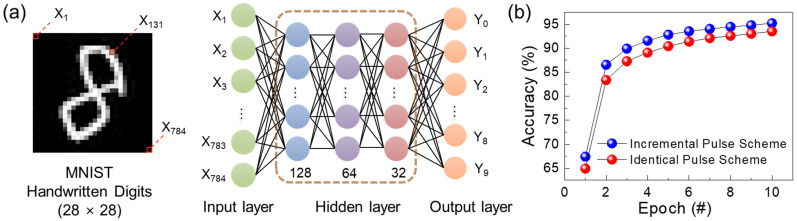
(**a**) Schematic of the artificial neural network designed for the MNIST simulation. (**b**) Pattern recognition accuracy as a function of the epoch. The data points in (**b**) were obtained from the MNIST simulation using the experimental LTP/LTD data shown in Figure 8.

As noted above, the linearity and symmetricity of the LTP/LTD characteristics directly affect the learning accuracy and training efficiency of the synapse. To assess the impact of these characteristics on image pattern recognition accuracy, we performed a theoretical simulation using the Modified National Institute of Standard and Technology (MNIST) handwritten digit dataset. The MNIST simulation was based on the backpropagation learning rule in an artificial neural network system, which includes 60,000 and 10,000 handwritten training and testing images, respectively. For this simulation, we assumed that the neural network consisted of a synthetic multilayer structure, including one input, three hidden, and one output layers (Figure 9a). Each training image of a handwritten digit was designed as a 28 × 28 pixel grid, converted into 784 input neuron vectors for the input layer. These input vectors were propagated through the three hidden layers (128 → 64 → 32 nodes) to the 10 output neurons. Based on updated synaptic weights for each test image, the pattern recognition accuracy was determined at the output layer by comparing the actual database values with the predicted output value. Then, the overall accuracy for all the test images was calculated as a percentage of the correct prediction by matching and comparing the predicted values with the true values. Through multiple runs of the MNIST simulation using the experimental data from Figure 8a,b, we found that the incremental pulse scheme achieved higher recognition accuracy than the identical pulse scheme (Figure 9b). For example, the pattern recognition accuracy increased from 93.5% (using the identical pulse scheme at 10 epochs) to 95.2% (using the incremental pulse scheme at 10 epochs). These results confirm that higher accuracy can be achieved when symmetric and linear LTP/LTD data are introduced to the neural network.

**Figure 10:**
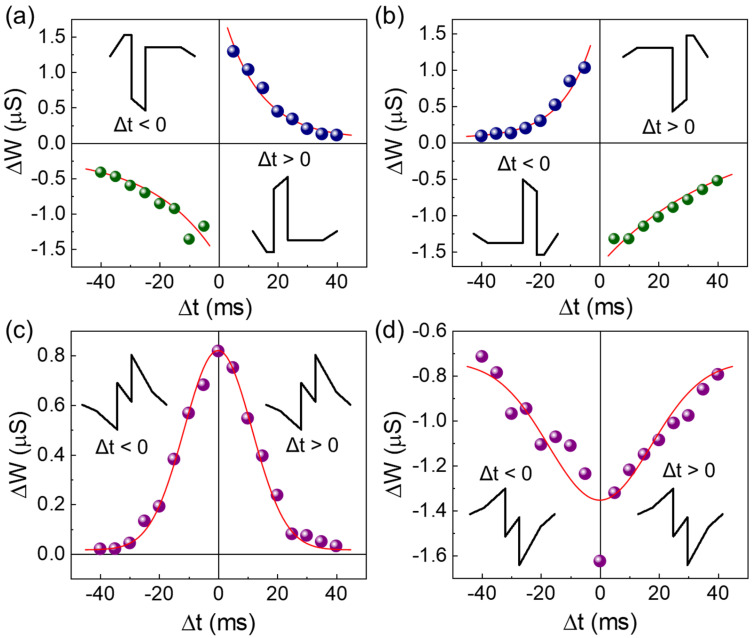
STDP characteristics of the Al/LiNbO_3_/Pt memristive synapse (LN-250), demonstrating the versatile learning activities of (**a**) asymmetric Hebbian, (**b**) asymmetric anti-Hebbian, (**c**) symmetric Hebbian, and (**d**) symmetric anti-Hebbian rules. Each inset shows the spike pulse scheme used for performing each Hebbian rule.

Finally, to examine the perceptron role of the LN-250 memristor as an electronic synapse, we measured its spike-timing-dependent plasticity (STDP) characteristics. In an electronic synapse, the perceptron role can be identified by observing the temporal difference between pre- and postsynaptic states [86,87,88]. The STDP measurement allows us to determine the synaptic weight change (Δ*w*) by varying the timing difference between pre- and postsynaptic spike pulses (i.e., Δ*t* = *t*_post_ − *t*_pre_). The variation of Δ*t*-dependent Δ*w* is typically used to assess the perceptron role of the electronic synapse. As shown in Figure 10, the LN-250 memristor successfully demonstrated four different types of Hebbian learning rules. Specifically, the asymmetric Hebbian (Figure 10a), asymmetric anti-Hebbian (Figure 10b), symmetric Hebbian (Figure 10c), and symmetric anti-Hebbian (Figure 10d) rules were realized by varying the polarity and/or shape of the applied spike pulses (see Figures S4–S7 for detailed Δ*t*-dependent spike pulse shapes). As seen in Figure 10a–d, in all four cases, Δ*w* decays exponentially with increasing Δ*t*. From the Δ*t*-dependent Δ*w* decay curves, the STDP time constant (*τ*_s_) can be parametrized using the following equations [89]:

(5) $$∆w=A\cdot exp\left(-\frac{\Delta t^{2}}{\tau_{s}^{2}}\right)+∆w_{0}\left(\text{for symmetric Hebbian rules}\right)$$

(6) $$∆w=A\cdot exp\left(-\frac{\Delta t}{\tau_{s}}\right)+∆w_{0}\left(\text{for asymmetric Hebbian rules}\right)$$

where *A* is the scaling factor, and Δ*w*_0_ is the constant synaptic weight that is independent of Δ*t*-dependent Δ*w*. By fitting the experimental data to Equations (5) and (6), the *τ*_s_ values were estimated to be 21.63, 40.26, 16.21, and 24.58 ms for the asymmetric Hebbian, asymmetric anti-Hebbian, symmetric Hebbian, and symmetric anti-Hebbian cases, respectively. These values fall within the timescale typical for biological synapses in the human brain (i.e., *τ*_s_ ≈ a few tens of milliseconds) [90]. Furthermore, since rapid Δ*w* changes within a narrow Δ*t* timescale are essential for parallel computing in neural networks, clear decay of Δ*t*-dependent Δ*w* is advantageous for future neuromorphic circuit applications. In summary, the present Au/LiNbO_3_/Pt memristive synapse demonstrated excellent functionalities as an electronic synapse, having comparable and even better synaptic performance than other V_O_-VCM-based memristive synapses (See Table 1).

## 4. Conclusions

The biological synaptic functions were effectively emulated using a memristive synapse, consisting of a top-to-bottom Al/LiNbO_3_/Pt two-terminal device that operates based on the V_O_-VCM mechanism. The device was fabricated by directly depositing a rhombohedral (113) LiNbO_3_ active layer onto a cubic (111) Pt bottom electrode, followed by the formation of a lithographic Al top electrode. The presence of V_O_ enabled V_O_-VCM-mediated SCLC in the LiNbO_3_ active layer, resulting in rectified asymmetric hysteresis characteristics. Furthermore, the device successfully demonstrated a range of synaptic functions by manipulating multiple memory states through control of the magnitude of *V*_pulse_ and the width of *t*_pulse_. It achieved an image pattern recognition accuracy of up to 95.2% in the MNIST simulation and exhibited versatile Hebbian learning behaviors in its STDP characteristics. These results highlight the potential of the V_O_-VCM-based Al/LiNbO_3_/Pt memristor for neuromorphic computing applications.

## Figures and Tables

**Table 1:** Comparison of materials and synaptic parameters for V_O_-VCM-based memristive synapse.

Materials	Pulse Condition for LTP and LTD				Dynamic Range	Linearity (LTP/LTD)	Pattern Recognition Accuracy	Ref.
	Pulse Scheme	*V*_LTP_/*V*_LTD_	*t*_LTP_/*t*_LTD_	*t*_inter_				
Pt/HfO_2_/HfO_x_/TiN	Identical	–0.8 V/2 V	1 μs/2 μs		~0.3~0.7 mA			[28]
TiN/Al:HfO_2_/TiN	Identical	2.5 V/–2.4 V	100 μs		3~9 μS	22%/60%	94.5%	[29]
Au/TiO_2_/Au	Identical	10 V/–10 V	50 ms	50 ms	~0.1~1 μA			[31]
ITO/TiO_x_/TiO_y_/TiN	Identical	1 V/–1 V	50 μs		~240~47 μA	0.89/0.69		[32]
W/WO_3−x_/Pt	Identical	1.8 V/–1.8 V	400 μs		~28~32 mA	0.81		[34]
Pt/Ta_2_O_5_/HfO_2_/TiN	Incremental	0.8~–1.2 V/1~1.2 V	10 μs		0~6 mS	27.03%/27.23%	69.88	[35]
Au/LiNbO_3_/Pt	Identical	+15 V/–15 V	100 ms		~17~23 μA			[37]
Au/LiNbO_3_/Pt	Identical	4 V/–4 V	40 ms	50 ms	~22~29 μA	1.2/2.7		[36]
This Work	Incremental	2~4.5 V/–0.5~3.5 V	300 μs/500 μs	10 ms	0~2 μA	0.16/0.32	95.2%	

## Data Availability

Data are contained within the article and Supplementary Materials.

## Supplementary Materials

The following supporting information can be downloaded at https://www.mdpi.com/article/10.3390/nano14231884/s1: Figure S1. (a) I–V characteristic curves of the Al/LiNbO_3_/Pt memristive devices composed of the (a–c) LN-180, (d–f) LN-250, and (g–i) LN-320 layers. Figure S2. (a) Schottky plot, (b) Fowler–Nordheim plot, (c) SCLC plot, and (d) Poole–Flenkel plot at the negative bias voltage region for the LN-250 memristive synapse. Figure S3. Dependence of ΔPSC on tpulse performed at the LTP and LTD operations: (a) *t*_pulse_ = 200 μs for LTP, (b) *t*_pulse_ = 400 μs for LTP, (c) *t*_pulse_ = 600 μs for LTP, (d) *t*_pulse_ = 800 μs for LTP, (e) *t*_pulse_ = 1 ms for LTP, and (f) *t*_pulse_ = 1 ms for LTD. *V*_pulse_ were 4–4.5 V and −2–−3 for LTP and LPD, respectively. Figure S4. Applied pulse schemes for demonstrating the asymmetric Hebbian learning rule when (a) Δ*t* = −5 ms, (b) Δ*t* = −20 ms, (c) Δ*t* = −40 ms, (d) Δ*t* = +5 ms, (e) Δ*t* = +20 ms, and (f) Δ*t* = +40 ms. Figure S5. Applied pulse schemes for demonstrating the asymmetric anti-Hebbian learning rule when (a) Δ*t* = −5 ms, (b) Δ*t* = −20 ms, (c) Δ*t* = −40 ms, (d) Δ*t* = +5 ms, (e) Δ*t* = +20 ms, and (f) Δ*t* = +40 ms. Figure S6. Applied pulse schemes for demonstrating the symmetric Hebbian learning rule when (a) Δ*t* = −5 ms, (b) Δ*t* = −20 ms, (c) Δ*t* = −40 ms, (d) Δ*t* = +5 ms, (e) Δ*t* = +20 ms, and (f) Δ*t* = +40 ms. Figure S7. Applied pulse schemes for demonstrating the symmetric anti-Hebbian learning rule when (a) Δ*t* = −5 ms, (b) Δ*t* = −20 ms, (c) Δ*t* = −40 ms, (d) Δ*t* = +5 ms, (e) Δ*t* = +20 ms, and (f) Δ*t* = +40 ms.
