# Supplementary material for: High-Performance Memristive Synapse Based on Space-Charge-Limited Conduction in LiNbO3
Source: Nanomaterials (Basel). 2024 Nov 23;14(23):1884. doi: 10.3390/nano14231884 (PMC11642943; doi:10.3390/nano14231884)
Supplement: Supplementary file 1 [file nanomaterials-14-01884-s001.zip › nanomaterials-3289636-supplementary.pdf]

nanomaterials

MDPI

Supplementary Materials

High-Performance Memristive Synapse Based on Space-Charge-Limited Conduction in $LiNbO_3$

Youngmin Lee [1,2] and Sejoon Lee [1,2,*]

[1] Division of System Semiconductor, Dongguk University, Seoul 04620, Republic of Korea; ymlee@dongguk.edu
[2] Quantum-Functional Semiconductor Research Center, Dongguk University, Seoul 04620, Republic of Korea
* Correspondence: sejoon@dongguk.edu

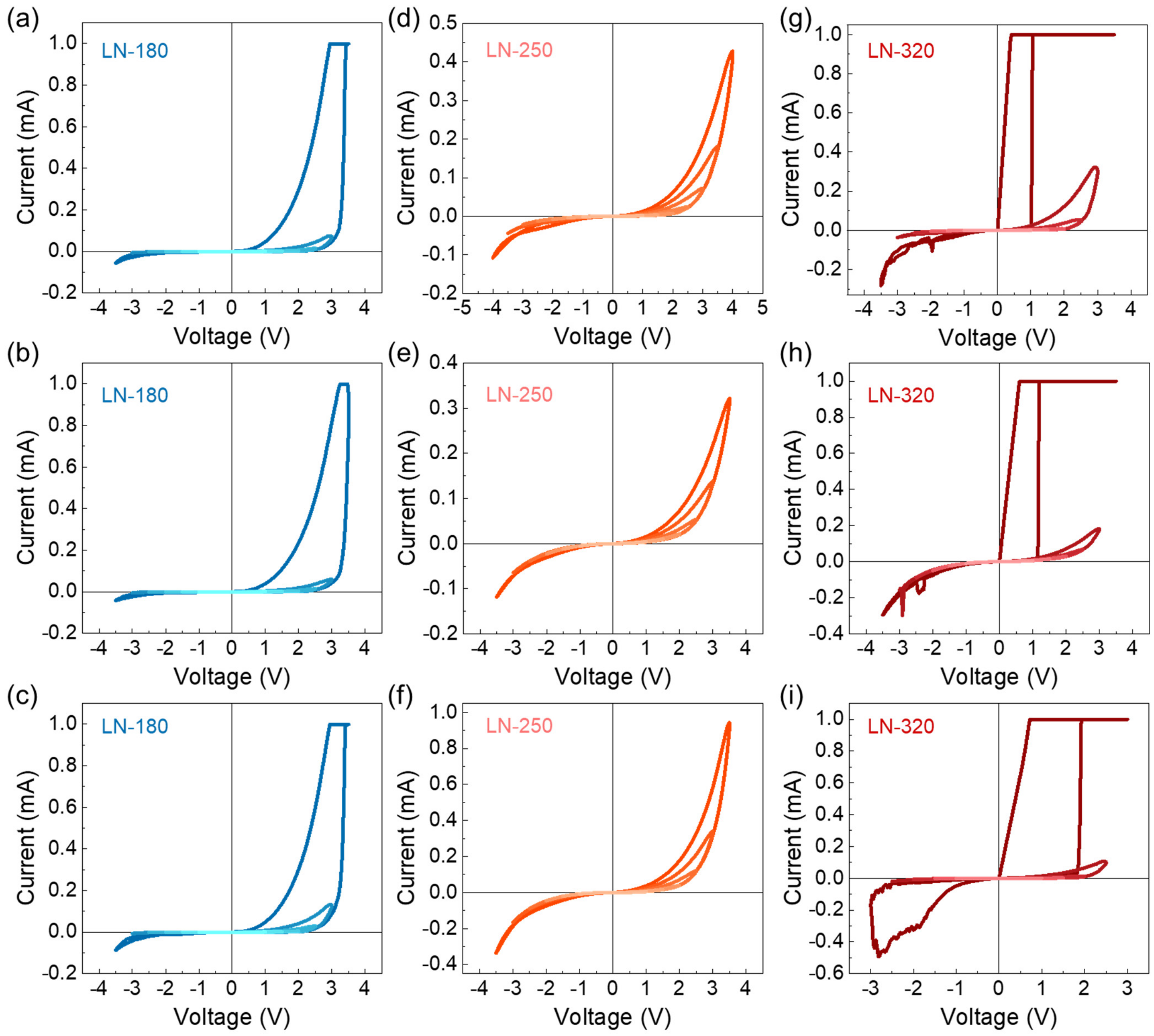

Figure S1. (a) I–V characteristic curves of the Al/$LiNbO_3$/Pt memristive devices composed of the (a)–(c) LN-180, (d)–(f) LN-250, and (g)–(i) LN-320 layers.

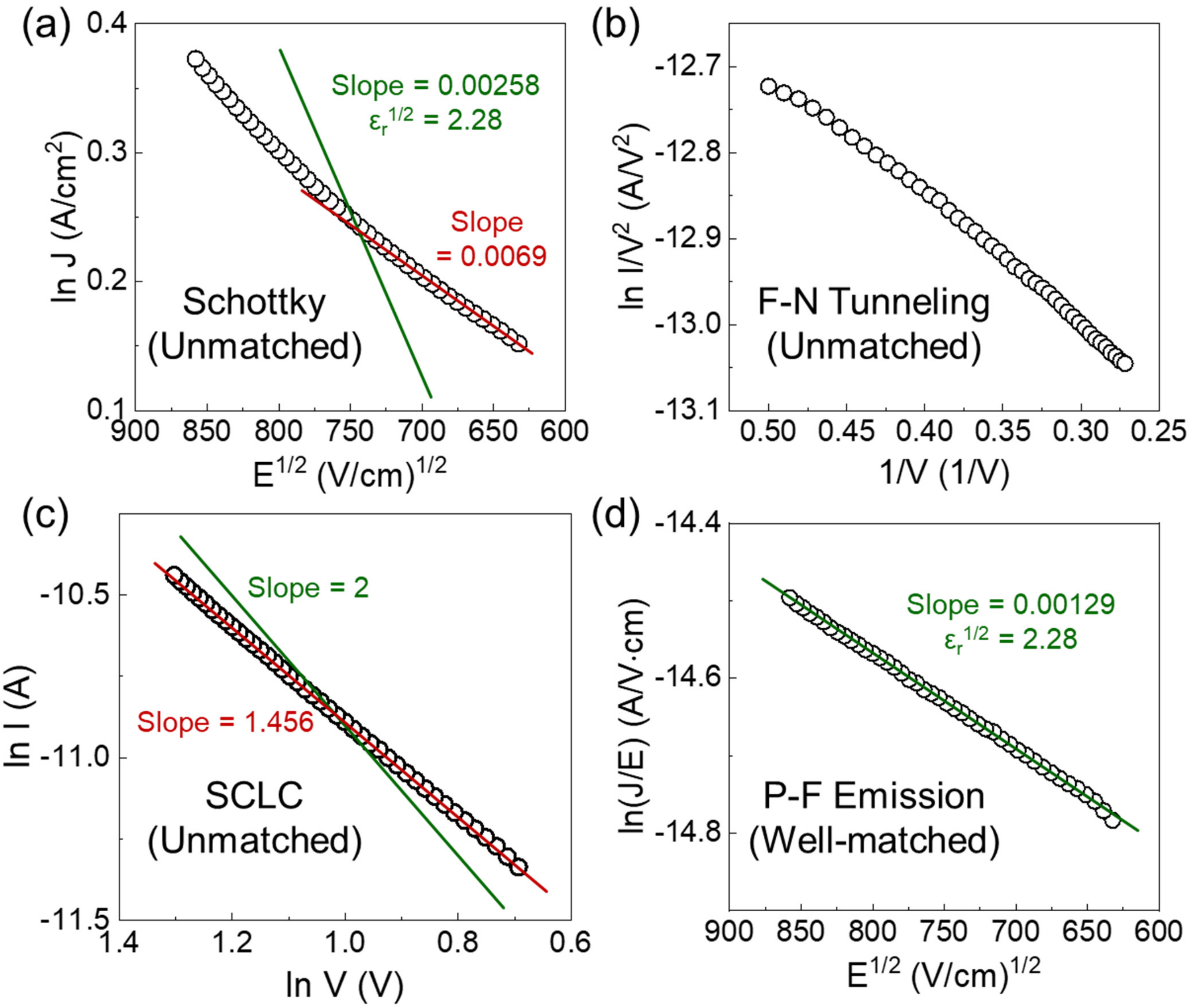

Figure S2. (a) Schottky plot, (b) Fowler-Nordheim plot, (c) SCLC plot, and (d) Poole-Flenkel plot at the negative bias voltage region for the LN-250 memristive synapse.

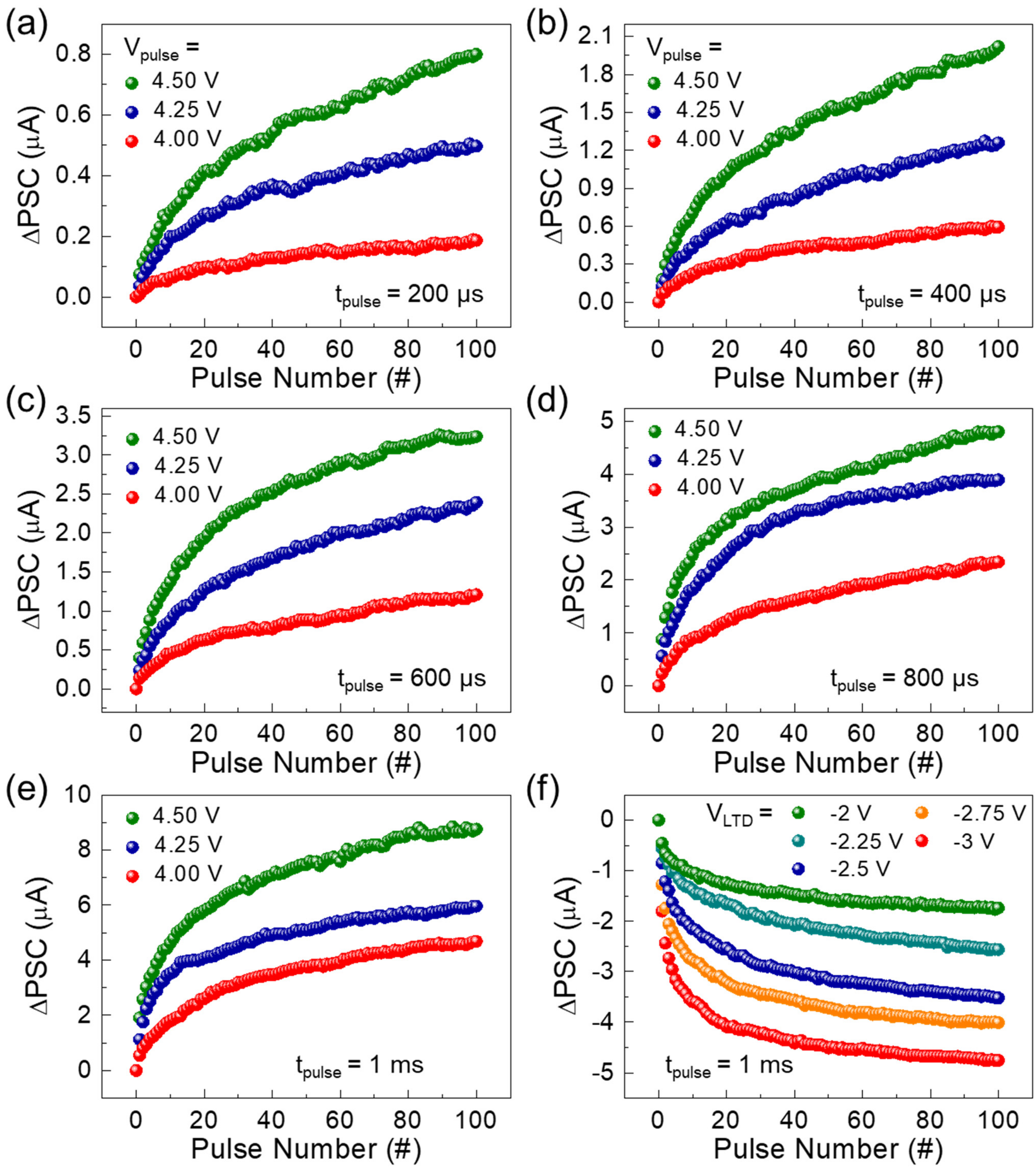

Figure S3. Dependence of ΔPSC on t_{pulse} performed at the LTP and LTD operations: (a) t_{pulse} = 200 μs for LTP, (b) t_{pulse} = 400 μs for LTP, (c) t_{pulse} = 600 μs for LTP, (d) t_{pulse} = 800 μs for LTP, (e) t_{pulse} = 1 ms for LTP, and (f) t_{pulse} = 1 ms for LTD. V_{pulse} were 4 – 4.5 V and −2 – −3 for LTP and LPD, respectively.

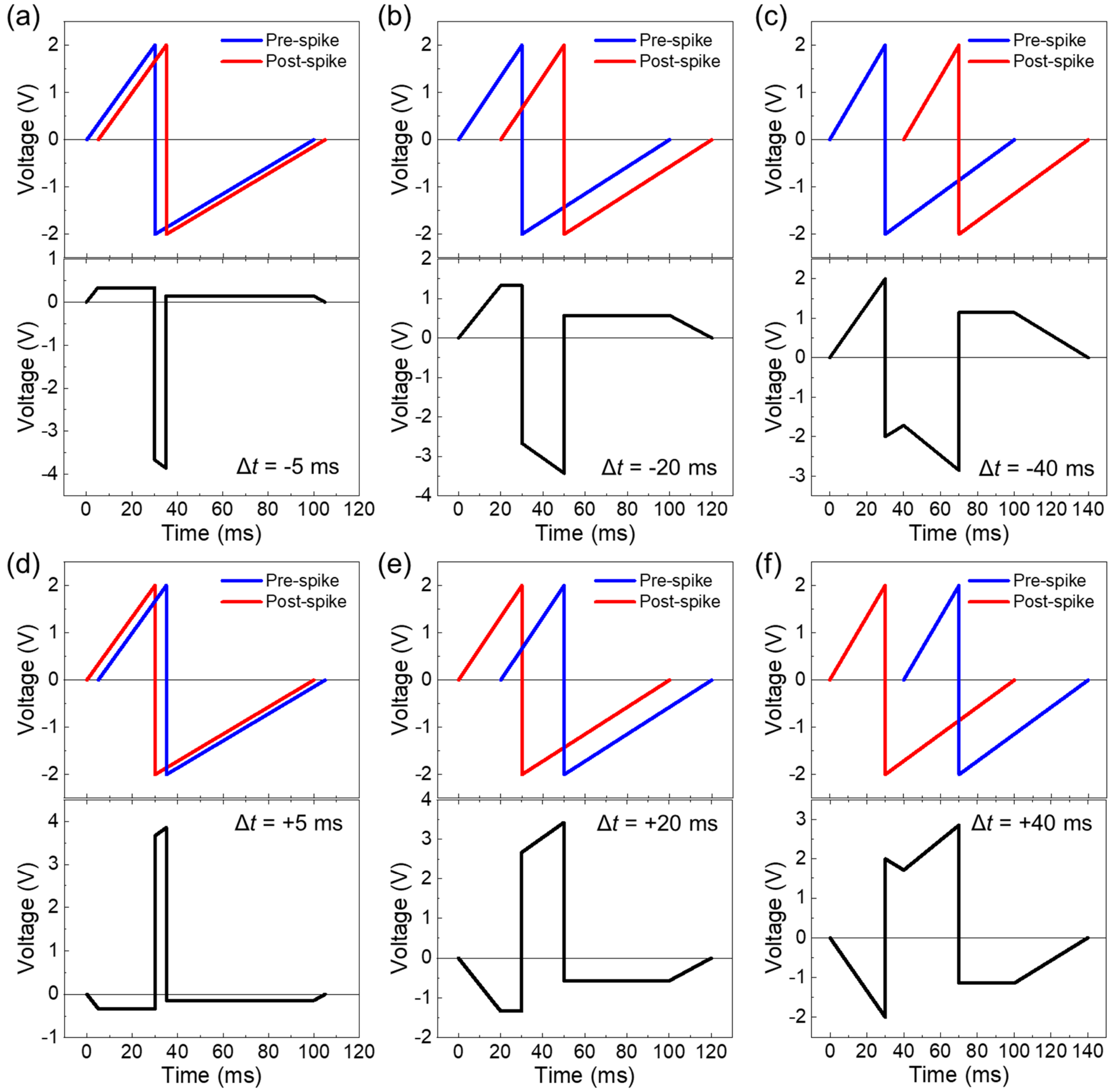

Figure S4. Applied pulse schemes for demonstrating the asymmetric Hebbian learning rule when (a) $\Delta t = -5$ ms, (b) $\Delta t = -20$ ms, (c) $\Delta t = -40$ ms, (d) $\Delta t = +5$ ms, (e) $\Delta t = +20$ ms, and (f) $\Delta t = +40$ ms.

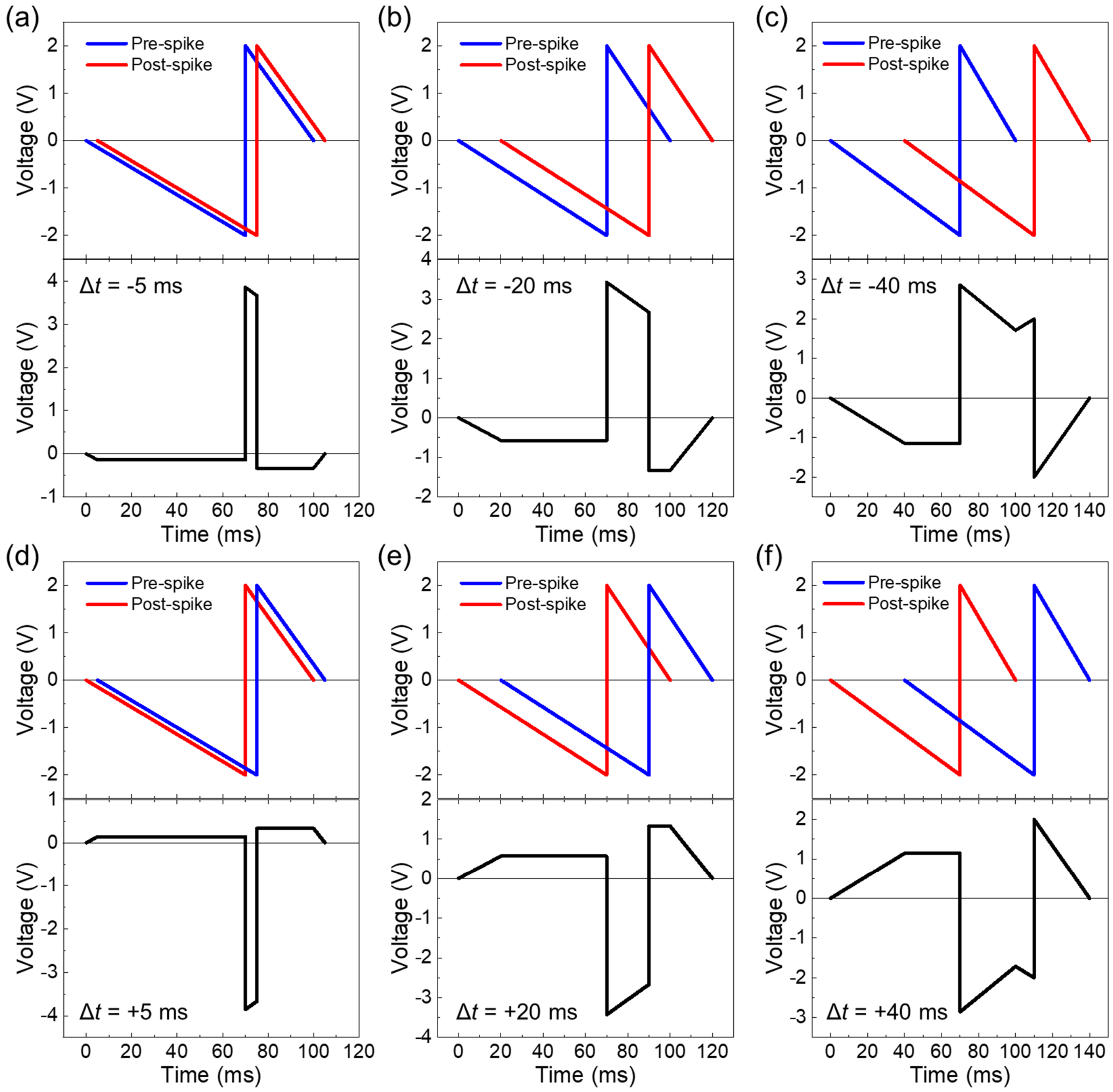

Figure S5. Applied pulse schemes for demonstrating the asymmetric anti-Hebbian learning rule when (a) Δt = −5 ms, (b) Δt = −20 ms, (c) Δt = −40 ms, (d) Δt = +5 ms, (e) Δt = +20 ms, and (f) Δt = +40 ms.

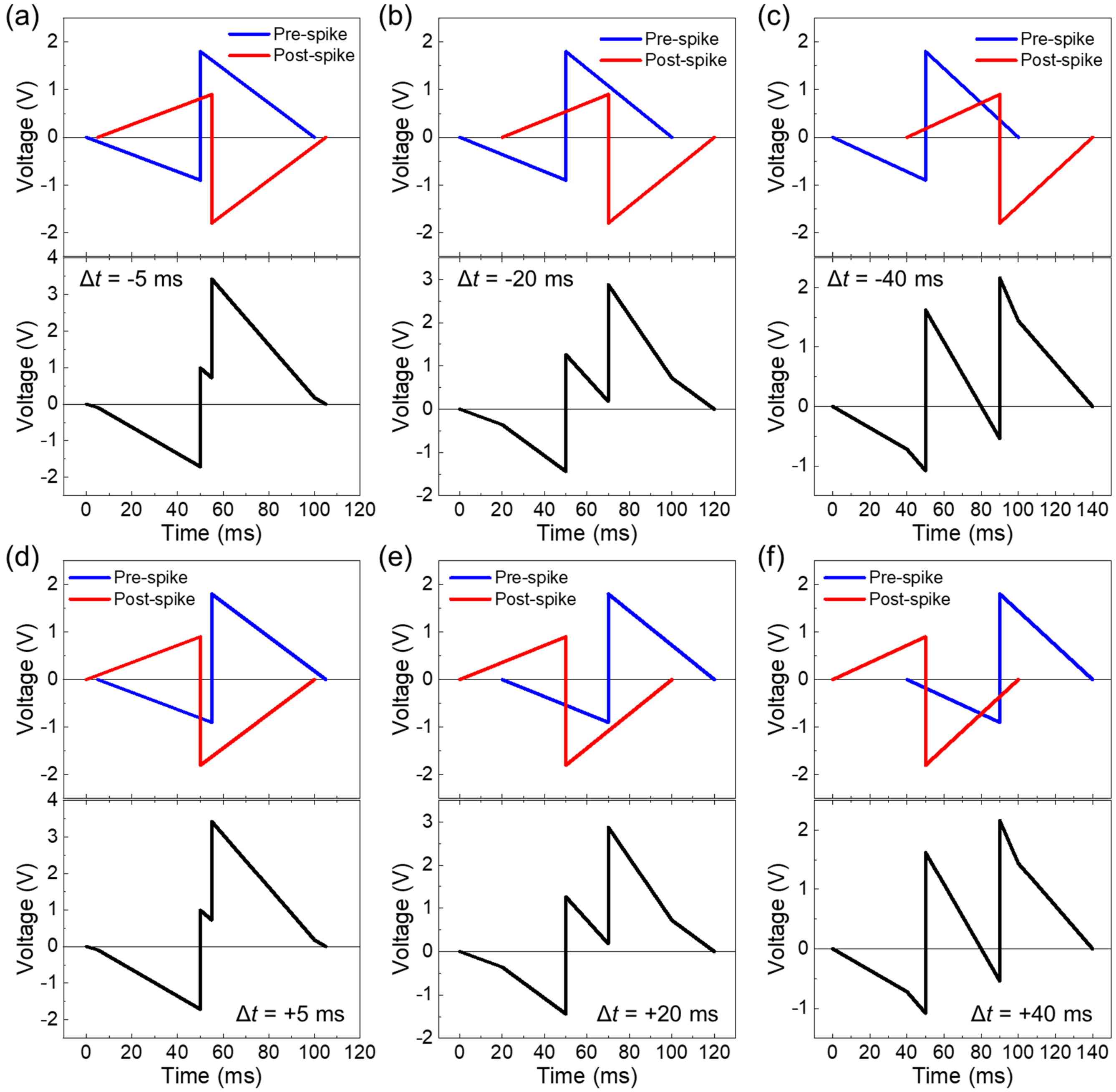

Figure S6. Applied pulse schemes for demonstrating the symmetric Hebbian learning rule when (a) Δ*t* = −5 ms, (b) Δ*t* = −20 ms, (c) Δ*t* = −40 ms, (d) Δ*t* = +5 ms, (e) Δ*t* = +20 ms, and (f) Δ*t* = +40 ms.

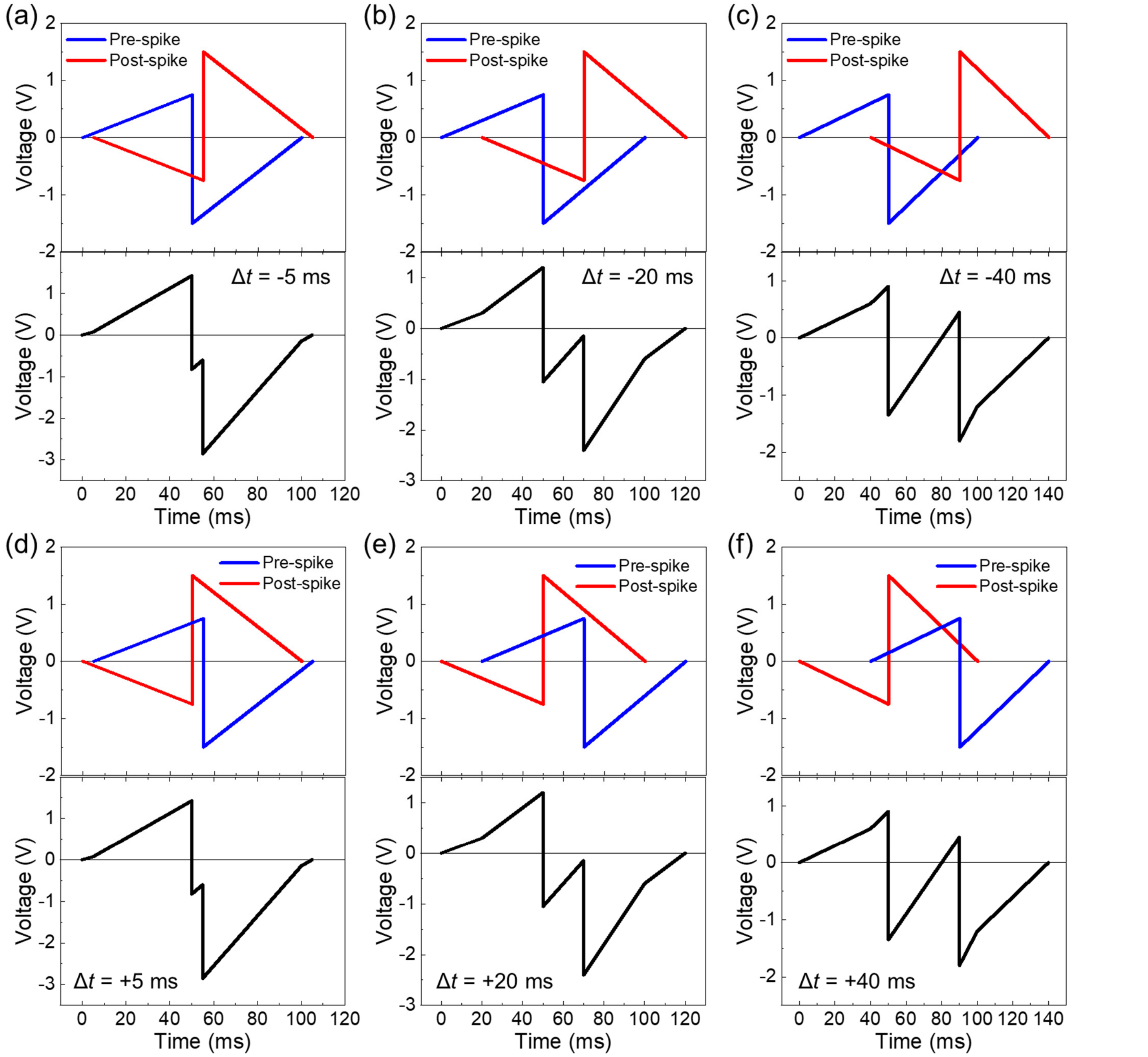

Figure S7. Applied pulse schemes for demonstrating the symmetric anti-Hebbian learning rule when (a) Δt = −5 ms, (b) Δt = −20 ms, (c) Δt = −40 ms, (d) Δt = +5 ms, (e) Δt = +20 ms, and (f) Δt = +40 ms.
